# Item analyses of the 20-item prosopagnosia index using classical test theory and item response theory

**DOI:** 10.3758/s13428-026-03145-3

**Published:** 2026-08-12

**Authors:** Masaki Mori, Midori Sugiyama

**Affiliations:** 1 Graduate School of Advanced Science and Technology, Japan Advanced Institute of Science and Technology, 1-1, Asahidai, Nomi, Ishikawa 923-1292 Japan; 2 Center for Data Science, Waseda University, 1-6-1, Nishi-Waseda, Shinjuku, Tokyo 169-8050 Japan; 3 Graduate School of Media and Governance, Keio University, 5322, Endo, Fujisawa, Kanagawa 252-0882 Japan

**Keywords:** Developmental prosopagnosia, Face processing, Item analyses, Classical test theory, Item response theory

## Abstract

The 20-item prosopagnosia index (PI20) is a highly practical tool for assessing lifelong difficulties in face processing across individuals (developmental prosopagnosia). Recent research suggests that the quality of the PI20 may vary depending on the item; however, the quantitative quality of each item has not been sufficiently examined. In this study, the item properties of the PI20 were investigated using classical test theory and item response theory. The analyses confirmed that the one-factor structure of the PI20 showed high reliability and validity. Item response theory further revealed that the items differed in measurement power, discrimination, and difficulty under the unidimensional graded response model. Items reflecting the additional psychological load and social consequences associated with face processing difficulty demonstrated high measurement precision. In contrast, items related to the recognition of distinctive or self-faces contributed to the construct to a lesser degree. These results indicate that while most items adequately capture the characteristics of developmental prosopagnosia, certain items may not be suitable for screening for developmental prosopagnosia. The present study provides open materials to facilitate further psychometric evaluation of the PI20 and offers insights for refining self-report assessment of developmental prosopagnosia.

## Introduction

Face processing is a crucial ability for identifying others in human social interactions. Nevertheless, face processing ability varies considerably across individuals (White and Burton, 2022). One manifestation of such individual differences is prosopagnosia, which is a selective impairment in the processing of familiar faces despite normal visual acuity, intelligence, and perception other than facial identity (Barton, 2008; Susilo and Duchaine, 2013). Prosopagnosia comprises two subtypes: acquired and developmental prosopagnosia. Acquired prosopagnosia is recognized as a higher-order cognitive dysfunction arising from brain injury, whereas developmental prosopagnosia is a neurodevelopmental property characterized by lifelong difficulties in face processing in the absence of overt brain injury (Bate and Tree, 2017; Duchaine and Nakayama, 2006b; Nørkær et al., 2024). Although developmental prosopagnosia is a famous topic in cognitive neuroscience, it lacks an official diagnostic category within major classifications such as the Diagnostic and Statistical Manual of Mental Disorders (American Psychiatric Association, 2022; Corrow et al., 2016; Gainotti, 2010). The prevalence rates of developmental prosopagnosia are reported to be 2.5% in Germany (n=689; Kennerknecht et al., 2006), 2.0% in China (n=8845; Zhao et al., 2018), 2.1%–2.9% in Australia (n=240; Bowles et al., 2009), and 0.1%–5.4% in the United States (n=3116; DeGutis et al., 2023). Given these high prevalence rates, calls for its inclusion in diagnostic frameworks have become more pronounced. However, research on developmental prosopagnosia suffers from the challenges in establishing diagnostic criteria, such as insufficient clarity in understanding its symptoms and a lack of precision in measurement (Bate and Tree, 2017; Burns, 2024; DeGutis et al., 2023; DeGutis and Campbell, 2024; Gerlach et al., 2024; Susilo and Duchaine, 2025). Therefore, identifying symptoms and establishing metrics are essential research topics in developmental prosopagnosia.

Measuring individual differences in face processing is crucial not only for developmental prosopagnosia but also for the theoretical understanding of face processing and the clinical assessment of individuals with strengths or difficulties in face processing. To achieve these ends, researchers have developed various experimental instruments that assess different stages of face processing. Several experimental instruments have been developed, and they can be broadly divided into perception-, recognition-, and identification-based tests (Robotham and Starrfelt, 2018). Perception-based tests focus on simultaneous discrimination and identity-matching processes for faces, such as the Cambridge Face Perception Test (Duchaine et al., 2007), Benton Facial Recognition Test (Benton and Van Allen, 1968; Murray et al., 2022; Rossion and Michel, 2018), Glasgow Face Matching Test (Burton et al., 2010; White et al., 2022), Kent Face Matching Test (Fysh and Bindemann, 2018), and Oxford Face Matching Test (Stantic et al., 2022). Recognition-based tests include the widely used the Cambridge Face Memory Test (Duchaine and Nakayama, 2006a; Russell et al., 2009), Warrington Recognition Memory for Faces Test (Warrington, 1984), and Ege Face Memory Test (Amado et al., 2025), which measure the ability of delayed discrimination and old/new face recognition. In identification, the Famous Faces Doppelgangers Test (Pozo et al., 2023), Famous Faces Recognition Test (Rizzo et al., 2002), and Italian Famous Face Test (Ventura et al., 2024) have also been employed to evaluate name-to-face matching and face naming, which rely on long-term and autobiographical memories for socially familiar identities. Together, these experimental measurement methods provide standardized and reproducible evaluations and are widely applied in research on individual differences in face processing.

Experimental measures of face processing ability tend to encounter several challenges, as they involve the use of face photographs as visual stimuli. One such challenge is the own-group bias in face processing. Faces differ in characteristics such as race and sex, and these attributes can influence face processing performance (Hugenberg et al., 2013; Meissner & Brigham, 2001; Wright & Sladden, 2003; cf., Herlitz & Lovén, 2013). Indeed, several face processing tests are designed to account for race and sex, and methods appropriate for each group are needed (Arrington et al., 2022; Kho et al., 2024; McKone et al., 2017). Nevertheless, considering all of these influences simultaneously remains a formidable challenge. Another challenge to overcome is reducing the time required for screening for developmental prosopagnosia and combining multiple tests. Experimental tasks typically last approximately 10 min, which is rather long for screening purposes. Furthermore, while combining multiple tests enhances accuracy, prolonging cognitively demanding tasks may decrease examinee performance. Therefore, simpler and fewer photograph-based screening tests are likely to result in greater social demand.

One of the most famous and widely used psychometric scales is the 20-item prosopagnosia index (PI20; Shah et al., 2015a). The PI20 is self-administered and has been reported to be a highly effective screening tool for developmental prosopagnosia (Tsantani et al., 2021). Indeed, the external validity of the PI20 has been confirmed using the Cambridge Face Memory Test (Estudillo & Wong, 2021; Gray et al., 2017; Livingston & Shah, 2018; Mejia et al., 2026; Nakashima et al., 2020; Nigrou et al., 2024; Nørkær et al., 2023; Shah et al., 2015a; Tagliente et al., 2023; Ventura et al., 2018), Famous Face Recognition Test (Livingston & Shah, 2018; Shah et al., 2015a; Ventura et al., 2018), the Cambridge Face Perception Test (Nørkær et al., 2023), Glasgow Face Matching Test (Shah et al., 2015b), and Italian Famous Face Test (Ventura et al., 2024).

Furthermore, the discriminant validity of the PI20 has been confirmed using the Cambridge Car Memory Test (Mejia et al., 2026; Nørkær et al., 2023; Shah et al., 2015a). Previous studies have reported that the PI20 can moderately measure developmental prosopagnosia traits in individuals with higher developmental prosopagnosia and the general population (Gray et al., 2017; Livingston and Shah, 2018). The PI20 has been developed and validated in the original version (English version; Shah et al., 2015a) and in at least nine other languages: Chinese (Sun et al., 2021), Danish (Nørkær et al., 2023), French (Nigrou et al., 2024), Italian (Tagliente et al., 2023), Japanese (Nakashima et al., 2020), Mandarin (Estudillo and Wong, 2021), Mexican Spanish (Mejia et al., 2026), Traditional Chinese (Wang et al., 2025) and Portuguese (Ventura et al., 2018). Thus, the PI20 enables high-quality screening surveys targeting a wide range of participants worldwide.

The PI20 was initially developed on the basis of expert knowledge, but several psychometric issues remain unresolved. For example, items with relatively low factor loadings (less than 0.40) have been identified, such as Items 3, 13, 14, 18, and 19 (Nakashima et al., 2020), as were Items 3 and 13 (Sun et al., 2021). Given that the PI20 is designed as a one-factor structure, such results indicate that these items exhibit low commonality (i.e., less than 0.16), which is calculated by the squared factor loading. This suggests that the quality and contribution of items within the PI20 are uneven, highlighting the need for further refinement of the scale. Indeed, several studies have raised concerns about the quality of the PI20 even though it is widely used (Arizpe et al., 2019; Matsuyoshi and Watanabe, 2021; Palermo et al., 2017). However, previous studies have not sufficiently examined the item properties of the PI20 and thus have not provided substantial quantitative insights into methods for improving the PI20. In particular, it is necessary to psychometrically identify which items are acceptable and inadequate.

To address the psychometric issues associated with the PI20, the present study examined item properties in a large sample using classical test theory and item response theory. Clarifying the properties of individual items provides the necessary discussion for future research aimed at improving the PI20 with fewer items or revising more effective items. Furthermore, previous studies have primarily examined convergent and discriminant validity (e.g., Nørkær et al., 2023; Shah et al., 2015a), and construct validity is inconsistent. Indeed, several previous studies have supported the one-factor structure of the PI20 through confirmatory factor analysis (Nakashima et al., 2020; Sun et al., 2021) with the exception of one study (Nørkær et al., 2023). Thus, this study confirmed whether the PI20 could clearly support a one-factor structure before examining the item properties specifically. Furthermore, this study provided large-scale open data and open code along with these verification results. These approaches signify the availability of a scheme for psychometrically evaluating the performance of an outstanding scale developed based on specialized knowledge primarily in the field of cognitive neuroscience. Thus, this study addressed the psychometric evaluation of the PI20, which has been relatively unexplored to date, thereby facilitating future research aimed at improving this scale.

## Methods

### Participants

The participants were 603 Japanese adults from the general population (302 females, 298 males, and 3 nonbinary; $M \pm SD = 42.97 \pm 10.10$ years). All the participants were recruited via a crowdsourcing service (CrowdWorks Inc.) and had a task approval rate of 95% or higher for the crowdsourcing service. All the participants correctly submitted a unique authentication code that was distributed to them after the survey ended. Four participants were excluded from the analysis based on two criteria: three participants responded incorrectly to one of the two items of the Directed Question Scale, and one participant did not agree to the instructions for appropriate responses (see Subsection“Instruments” for detailed procedures). Therefore, the data from 599 participants (298 females, 298 males, and three nonbinary; $M \pm SD = 42.97\pm 10.12$ years) were valid.

According to the simulation-based studies, n=300 is the minimum sample size required for the confirmatory factor analysis (Myers et al., 2011) and a graded response model (Dai et al., 2021). Considering the response quality in online surveys and the robustness of the estimated parameters, we recruited twice the number of participants compared to the minimum sample size. To the best of the authors’ knowledge, this study had the second largest number of participants following a previous study (n=647; Sun et al., 2021).

### Instruments

This study used the Japanese version of the PI20 (Nakashima et al., 2020). The reliability and validity of the Japanese version of the PI20 have been strongly evaluated in a previous study. The participants responded to each of the 20 items in the PI20 using a 5-point Likert scale (1: *strongly disagree*, 5: *strongly agree*). However, Items 8, 9, 13, 17, and 19 were semantically opposed to developmental prosopagnosia (reversed items); therefore, responses to 1, 2, 3, 4, and 5 were converted to scores of 5, 4, 3, 2, and 1, respectively. The total scores ranged from 20 to 100 points.

To ensure the quality of the online survey and prevent insufficient effort in response, this study added supplementary instructions for appropriate responses (Huang et al., 2012; Masuda et al., 2019) and two items from the Directed Question Scale (Maniaci and Rogge, 2014; Miura and Kobayashi, 2015) to the Japanese version of the PI20. The instructions for appropriate responses were presented immediately before the start of the responses for the PI20, and participants had the option to write a check mark. The items of the Directed Question Scale were presented as follows: “For this question, please select *strongly agree*.” was placed after Item 5, and “For this question, please select the top option.” was placed after Item 15. The presentation order and phrasing of Items 1–20 matched the Japanese version of the PI20.

### Procedure

This survey was created and conducted using an online survey software tool (Qualtrics). The online survey forms were distributed to registered users through a crowdsourcing service. Participants responded using their own electronic devices. To maintain attention control, participants responded to each item presented on the screen one by one. To avoid careless responses, participants could proceed to the next item only 2 s after each item was presented and only if they had responded. After all the responses were received, an eight-digit verification code displayed in Qualtrics must be entered accurately into the crowdsourcing service’s interface. The total response time for each participant was approximately 3 min. This survey was conducted from July 2025 to August 2025.

### Analyses

Analyses based on classical test theory were conducted to confirm the floor effect, ceiling effect, internal consistency, and the structural validity of the one-factor structure. The floor effects were evaluated by M-SD<1 for each item. Similarly, the ceiling effects were evaluated by M+SD>5. CITCs (corrected item-total correlations) were calculated to examine the consistency of each item with the overall PI20 score (with the item itself excluded from the total). CITC values exceeding .30 were regarded as acceptable, and those above .50 were taken to reflect strong consistency.

A good-poor analysis was conducted to evaluate the discrimination of each item. Participants were divided into the upper 27% (high-score group) and lower 27% (low-score group) based on the total score of the PI20. For each item, mean scores between the two groups were compared using independent-samples *t* tests. In addition to significance testing, Cohen’s *d* was calculated to evaluate the standardized magnitude of group differences. In accordance with Cohen ’s (1988) guidelines, d≥0.50 (medium) was considered indicative of adequate discrimination. Since the PI20 is regarded as having a one-factor structure (Nakashima et al., 2020; Sun et al., 2021; cf., Nørkær et al., 2023), the reliability and validity of the one-factor structure were evaluated. Internal consistency was evaluated by Cronbach’s α and McDonald’s ω. Structural validity was examined with confirmatory factor analysis, applying the robust weighted least squares estimator appropriate for categorical indicators. We computed the factor loadings ($\lambda _1, \lambda _2,...,\lambda _{20}$), residual terms ($e_1, e_2,...,e_{20}$), and model fit ($\chi ^2$ test, comparative fit index [CFI], Tucker–Lewis index [TLI], and root mean square error of approximation [RMSEA]) on the basis of a structural equation model to evaluate the validity of the unidimensional structure. Model fit was evaluated according to the criteria proposed by Hair et al. (2018): CMIN/DF<3, CFI>.95, TLI>.95, and RMSEA<.08.

When the results of the confirmatory factor analysis supported the unidimensionality of the PI20, unidimensional graded response model analysis (Samejima, 1969, 1997), which is an item response theory, was conducted to evaluate item discrimination and difficulty. Let $u_i$ be the category of the Likert scale ($u_i=\{1,2,..,5\}$) for Item *i* ($i=\{1,2,...,20\}$), and the response probability $P_{u_i}(\theta )$ of each item to the latent ability of developmental prosopagnosia (θ) was derived using an extended version of the two-parameter logistic model ($a, b_2, b_3, b_4,~\textrm{and}~b_5$), which approximates the normal ogive model with the scaling constant D=1.7. Local independence was evaluated by calculating $\mathrm {Yen's~}Q_{3}( {p,q}\mathrm {)}$ (*p* and *q* represent item numbers) and $\bar{Q}_{3}$ ($\frac{1}{190} \sum _{1 \le p < q \le 20} Q_{3}(p,q)$). There is no universally accepted single critical value for the $Q_3$ statistic (Christensen et al., 2017; Nason and Demars, 2025). We followed the guideline of considering that local independence cannot be assumed when these values exceed 0.30 (Aryadoust et al., 2021). The overall model fit of the unidimensional graded response model was assessed with the $M_2$ statistic, CFI, TLI, RMSEA, and standardized root mean square residual (SRMSR). Overall reliability was assessed on the basis of the marginal reliability coefficient ($\rho _\mathrm{{MR}}$). The item information curve illustrates the measurement precision provided by each item along the latent trait of developmental prosopagnosia (θ). The test information curve reflects the overall measurement precision of the scale across the range of θ. The interpretation of parameters and information curves followed the criteria proposed by Baker (2001): i.e., a≥1.70 is *very high discrimination*, $1.35\le {a}\le 1.69$ is *high discrimination*, $0.65\le {a}\le 1.34$ is *moderate discrimination*, $0.35\le {a}\le 0.64$ is *low discrimination*, a≤0.34 is *very low discrimination*, $-2.8\le a\le 2.8$ is *typical discrimination*, and $-3.0\le b\le 3.0$ is *typical difficulty*. The item parameters of the graded response model were estimated using marginal maximum likelihood with an expectation-maximization algorithm.

**Table 1:** Descriptive and item analysis results for the PI20 based on classical test theory

Item	Descriptive statistics				CITC	Good-poor analysis					
	*M*	*SD*	$g_1$	$g_2$		$M_\mathrm{{high}}$	$M_\mathrm{{low}}$	ΔM	*t*(*df*)	*p*	*d*
1	2.69	1.05	0.12	-0.63	0.72	3.69	1.71	1.98	23.94 (313)	<.001	2.67
2	2.74	1.20	0.25	-1.00	0.81	4.09	1.61	2.47	32.51 (314)	<.001	3.62
3	4.26	0.76	-1.02	1.21	-0.21	4.11	4.47	-0.36	-4.07(315)	<.001	-0.45
4	2.34	1.00	0.67	-0.11	0.74	3.41	1.53	1.88	22.76 (275)	<.001	2.54
5	1.93	1.01	1.09	0.66	0.75	3.02	1.14	1.88	21.94 (201)	<.001	2.44
6	3.05	1.07	-0.11	-0.87	0.66	3.97	2.14	1.83	20.29 (319)	<.001	2.26
7	1.83	1.00	1.15	0.65	0.74	2.93	1.07	1.86	22.51 (184)	<.001	2.51
8†	2.95	1.07	0.16	-0.73	0.64	3.86	2.08	1.78	18.57 (319)	<.001	2.07
9†	3.28	1.08	-0.08	-0.74	0.70	4.31	2.33	1.98	22.49 (314)	<.001	2.51
10	2.12	0.93	0.61	-0.22	0.70	3.06	1.32	1.75	22.27 (259)	<.001	2.48
11	1.92	1.10	1.07	0.20	0.72	3.07	1.08	1.99	21.52 (178)	<.001	2.40
12	2.34	1.21	0.61	-0.62	0.82	3.75	1.25	2.50	29.93 (240)	<.001	3.34
13†	1.73	0.89	1.16	0.83	0.36	2.19	1.25	0.94	10.00 (222)	<.001	1.11
14	2.31	1.18	0.54	-0.78	0.59	3.25	1.42	1.83	17.21 (270)	<.001	1.92
15	1.85	0.99	1.03	0.36	0.70	2.89	1.10	1.79	22.12 (190)	<.001	2.47
16	2.06	1.08	0.80	-0.26	0.70	3.17	1.21	1.96	22.86 (241)	<.001	2.55
17†	2.90	1.11	0.06	-0.78	0.74	3.99	1.81	2.18	25.42 (320)	<.001	2.83
18	1.84	1.05	1.15	0.37	0.56	2.71	1.19	1.52	15.19 (213)	<.001	1.69
19†	3.54	1.02	-0.34	-0.43	0.54	4.25	2.78	1.48	15.30 (299)	<.001	1.71
20	2.63	1.13	0.28	-0.84	0.70	3.71	1.66	2.05	22.45 (300)	<.001	2.50

*Notes.* *M* = mean; *SD* = standard deviation; $g_1$ = skewness; $g_2$ = kurtosis; CITC = corrected item-total correlation; $M_\textrm{high}$ = mean score of the upper 27% group; $M_\textrm{low}$ = mean score of the lower 27% group; ΔM = $M_\textrm{high}-M_\textrm{low}$; *t*(*df*) = *t* value with degrees of freedom; *p* = *p* value; *d* = Cohen’s *d*. The symbol † indicates a reversed item; therefore, the score for each item reflects the corrected value

These analyses were performed using R (version 4.5.1; R Core Team, 2025) with tseries (version 0.10.58; Trapletti et al., 2025), e1071 (version 1.7.16; Meyer et al., 2024), lavaan (version 0.6.19; Rosseel, 2012), psych (version 2.5.6; Revelle, 2025), semTools (version 0.5.7; Jorgensen et al., 2025), and mirt (version 1.44.0; Chalmers, 2012). The significance level was set at 5% for the hypothetical statistical analysis.

**Fig. 1:**
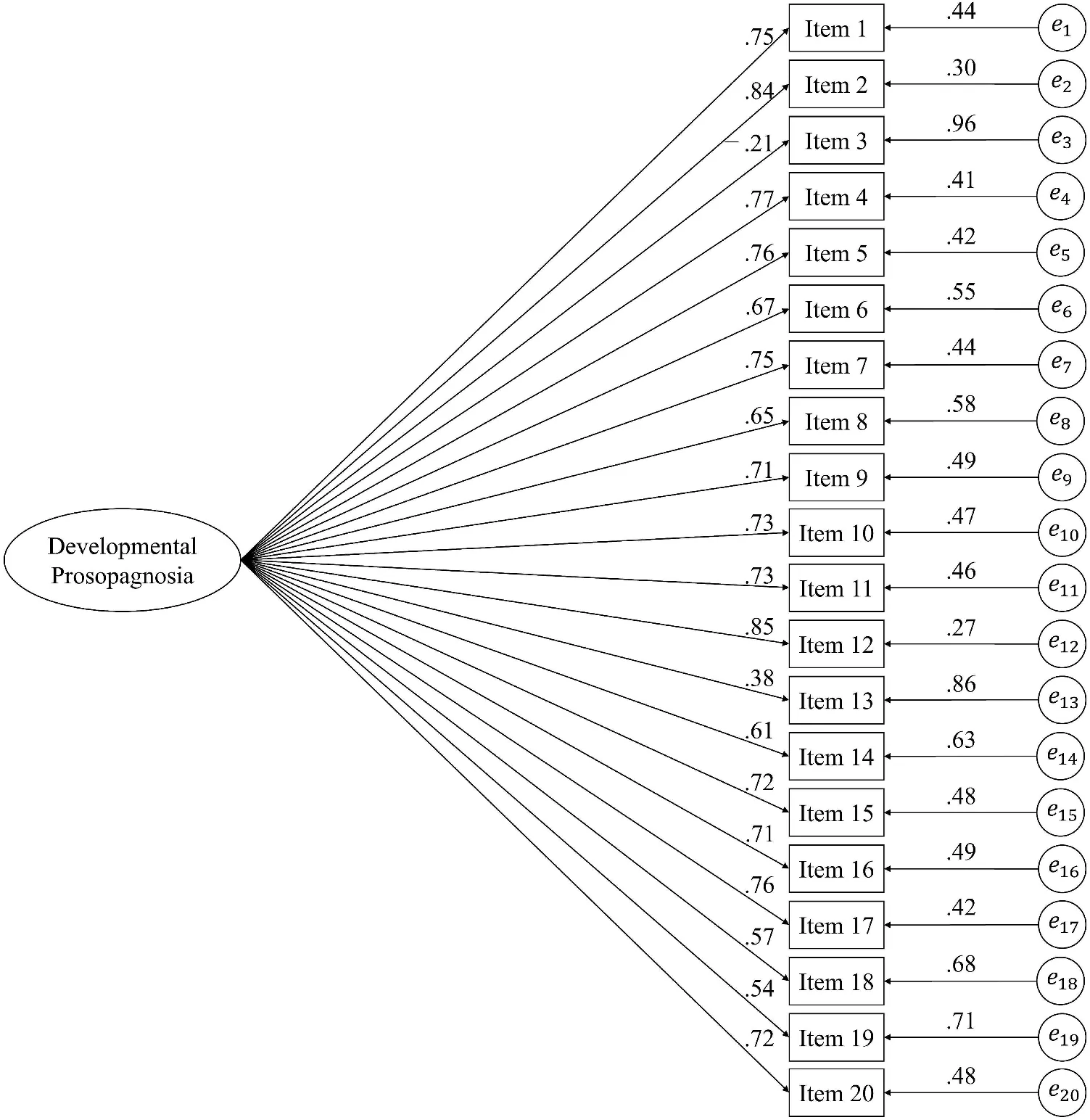
Standardized path diagram from the confirmatory factor analysis of the PI20

**Fig. 2:**
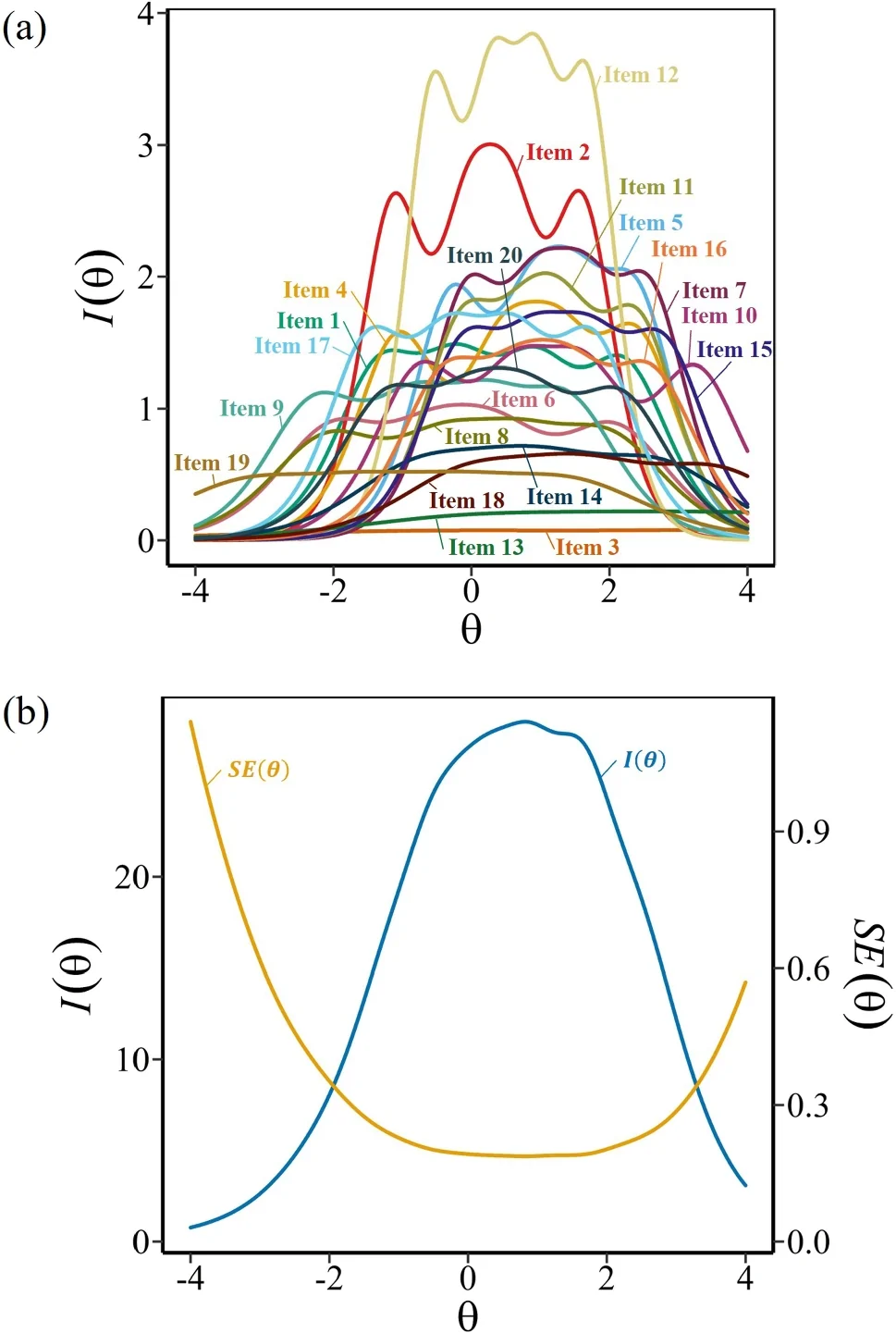
Information curves of the PI20. (**a**) Item information curves. (**b**) Test information curve. *Notes.* θ denotes the latent trait of developmental prosopagnosia. (**a**) I(θ) shows the amount of information for each item. (**b**) The curves of I(θ) and SE(θ) represent the test information and the standard error of measurement, respectively

## Results

### Classical test theory analyses

We first conducted descriptive analyses to evaluate the item-level properties of each item. Descriptive and item analysis results for the valid data are shown in Table 1. Most items showed no evidence of floor or ceiling effects, indicating adequate variability across the response scale. Specifically, all the items except for Items 5, 7, 11, 13, 15, 16, and 18 showed no floor effect, and all the items except for Item 3 showed no ceiling effect. All the items except for Items 3 and 13 had CITCs exceeding .50, indicating strong internal coherence among items. The CITC for Item 13 was acceptable, whereas that for Item 3 was notably low, suggesting that this item contributed less to the overall construct. In the good-poor analysis, significant differences were observed between the high- and low-score groups for all items. For Items 1, 2, and 4–20, the high-score group scored higher than the low-score group did, whereas Item 3 showed the opposite pattern. With the exception of Item 3 (d=-0.45), all the items exhibited strong discriminative power, with effect sizes ranging from Cohen’s d=1.11 to 3.62.

Overall, the internal consistency coefficients for the PI20 were high, with Cronbach’s α=.94 and McDonald’s ω=.95, confirming that the PI20 items consistently measure a common latent construct.

Confirmatory factor analysis was conducted to evaluate the model fit of the unidimensional structure. A standardized path diagram illustrating the one-factor structure of the PI20 is shown in Fig. 1. The standardized path coefficients exceeded .40 for all the items except Items 3 and 13, indicating that most of the items were meaningfully associated with the latent construct (developmental prosopagnosia). The analysis yielded $\chi ^2(170) = 298.49$, $p <.001, \mathrm {CMIN/DF}=1.76$, $\textrm{CFI}=.99$, $\textrm{TLI}=.99$, and $\textrm{RMSEA}=.04$. When the sample size is large, the chi-squared test detects even slight discrepancies from the true model. Except for the results of the chi-squared test, all indices demonstrated model fit satisfying the criteria proposed by Hair et al. (2018).

### Item response theory analyses

To evaluate local independence before model evaluation and parameter estimation, we computed $Q_3$ statistics for all item pairs and the $\bar{Q}_{3}$ statistic for the overall scale. The $\bar{Q}_{3}$ statistics was -.04, and all the item pairs had $|Q_{3}|$ statistics less than .30 ($-.27\le Q_{3}\le .29$). On the basis of these results, the PI20 was considered to meet the local independence assumption, and the subsequent analyses were conducted accordingly. In summary, the results supported the validity of further model evaluation.

To examine the overall model fit of the unidimensional graded response model, we evaluated several goodness-of-fit indices. Although the $M_2$ statistic was statistically significant, $M_2(110) = 255.86, p <.001$, the other indices indicated good model fit, $\textrm{CFI}=.97$, $\textrm{TLI}=.96$, $\textrm{RMSEA}=.05$, and $\textrm{SRMSR}=.05$. Given the large sample size, the significant $M_2$ test may reflect trivial poor fit rather than substantive model inadequacy. Therefore, we hold that this model demonstrates a sufficiently acceptable fit. The marginal reliability of the PI20 was high at $\rho _\mathrm{{MR}}=.96$. Overall, these findings support the validity and reliability of the unidimensional graded response model for the PI20.

To further explore item-level and overall scale performance, the curves of item and test information for the latent traits of developmental prosopagnosia are shown in Fig. 2. The item information curve peaks were distributed across a wide range of θ values, indicating that the item set provides measurement precision for respondents from low to high latent trait levels. In particular, Items 2, 4, 5, 7, 11, 12, 15, and 17 exhibited high information peaks, reflecting strong discrimination power, whereas Items 3 and 13 contributed relatively little information across the θ continuum. Items 2, 4, and 17 provided a great amount of information within the approximate range of -2<θ<2. Item 12 showed peak information between approximately -1 and 2 on θ. Items 5, 7, 11, and 15 contributed most strongly within the approximate range of 0<θ<3. Figure 2b shows the test information curve. The TIC peaked around θ=0-2, indicating that the test provides the highest precision for respondents with average to moderately high levels of the trait. Adequate information ($I(\theta ) \le 5$) was found across the range of -2<θ<3, suggesting that the PI20 provided a reliable measurement within this interval. Taken together, these results indicate that the PI20 offers precise measurement of developmental prosopagnosia traits across a broad range of ability levels, particularly around the moderate to high range.

**Fig. 3:**
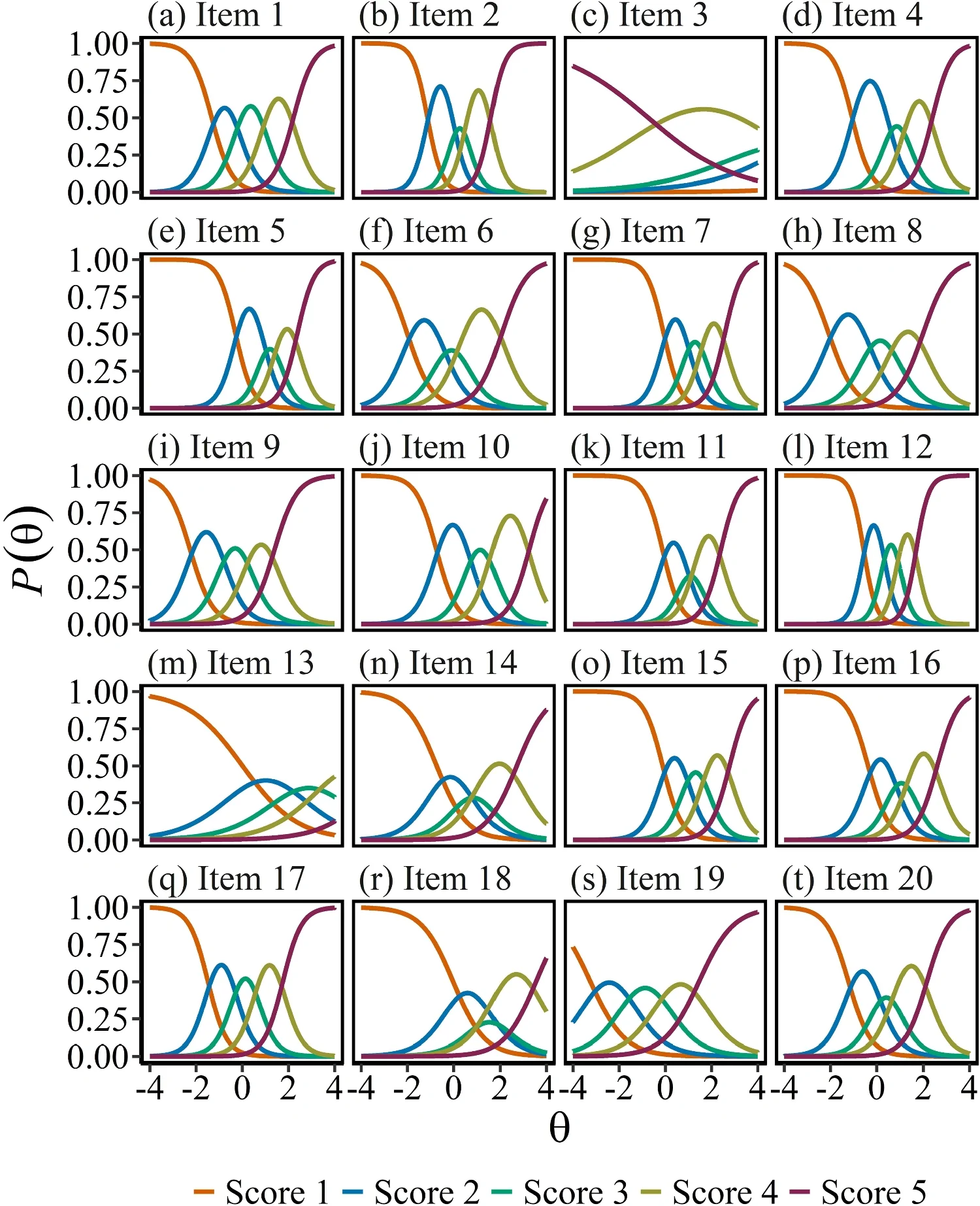
Item response category characteristic curve of PI20. (**a**)–(t) correspond to Items 1–20, respectively. *Notes.* θ denotes the latent trait of developmental prosopagnosia. P(θ) indicates the probability of each score ($u_i = 1, 2,..., 5$) as a function of θ

To interpret the item characteristics in detail, the item response category characteristic curves of the PI20 are shown in Fig. 3. The parameters of item discrimination (*a*) and difficulty ($b_2, b_3, b_4,~\textrm{and}~b_5$) for each function of latent traits (θ) are shown in Table 2. In accordance with the criteria proposed by Baker (2001), the discrimination (*a*) of all the items was within the standard range. Items 2 and 12 had very high levels of discrimination. Items 1, 4, 5, 7, 11, 15, and 17 had high levels of discrimination. Items 6, 8, 9, 10, 14, 16, 18, 19, and 20 had moderate discrimination. Item 13 had low discrimination. Item 3 had very low discrimination. Furthermore, the difficulty (*b*) of Items 1, 2, 4, 5, 6, 7, 8, 9, 11, 12, 14, 15, 16, 17, and 20 was within the standard range. However, the $b_1$ for Item 3, $b_2$ for Item 3, $b_3$ for Items 3 and 13, and $b_4$ for Items 10, 13, and 18 were greater than 3. $b_1$ in Item 19 was less than -3. The results for these items suggest that the difficulty level is either excessively high or excessively low. These results suggest that certain items (particularly Items 3 and 13) exhibited extreme difficulty parameters, indicating potential issues with their response range. In sum, the item parameter estimates indicate that most items function appropriately, although Items 3 and 13 demonstrate limited discrimination and atypical difficulty levels.

## Discussion

The present study aimed to evaluate the psychometric properties of the PI20. Using classical test theory and item response theory analyses, this study revealed that most items contribute meaningfully to measuring developmental prosopagnosia traits. In particular, Items 2, 4, 5, 7, 11, 12, 15, and 17 provided excellent discrimination, difficulty, and information. Nevertheless, Items 3 and 13 consistently showed weaker psychometric properties, such as insufficient discrimination and difficulty. These findings suggest that while the PI20 performs well as a measurement tool for most items, certain items could be revised to improve measurement accuracy.

This study indicated that the PI20 demonstrated high internal consistency and acceptable unidimensional model fit on the basis of the results of the confirmatory factor analysis, which is consistent with previous findings (Nakashima et al., 2020; Nørkær et al., 2023; Sun et al., 2021). In addition, this study revealed that the PI20 was well represented by a unidimensional graded response model through reliability and validity analysis in item response theory. These results support the validity of the unidimensional assumption, suggesting that the PI20 reliably captures a single latent construct of developmental prosopagnosia.

Items 2, 4, 5, 7, 11, 12, 15, and 17 showed excellent performance in terms of item information, discrimination, and difficulty. These items (e.g., Item 5: “*When I was at school, I struggled to recognize my classmates*”; Item 12: “*I have to try harder than other people to memorize faces*”) may share the common feature of reflecting the additional psychological load and social consequences associated with difficulties in recognizing facial identity, which in cognitive models of face processing have been linked to impaired access to face recognition units and person identity nodes (Bruce & Young, 1986; Duchaine & Yovel, 2015). Previous studies have repeatedly noted that the time required for face processing may be a crucial indicator of face processing difficulty (Malaspina et al., 2018; Murray et al., 2022; Rossion and Michel, 2018). Taking longer to process faces may not simply reflect a delay in processing speed, but may also indicate a psychological load regarding attentional resources and social interaction. Furthermore, face processing difficulty significantly affects social life (Dalrymple et al., 2014; Yardley et al., 2008), skills (Adams et al., 2020; Bate et al., 2010), and anxiety (Davis et al., 2011). Therefore, these item characteristics may represent the essential difficulty features of developmental prosopagnosia.

**Table 2:** Estimated parameters of the unidimensional graded response model for the PI20

Item	*a* (SE)	$b_2$ (SE)	$b_3$ (SE)	$b_4$ (SE)	$b_5$ (SE)
1	1.36 (0.09)	-1.32(0.09)	-0.21(0.06)	0.93 (0.08)	2.21 (0.14)
2	1.88 (0.12)	-1.13(0.08)	-0.02(0.06)	0.55 (0.06)	1.60 (0.09)
3	-0.31(0.05)	12.42 (2.81)	6.54 (1.14)	4.06 (0.69)	-0.74(0.20)
4	1.46 (0.10)	-1.06(0.08)	0.49 (0.06)	1.25 (0.08)	2.39 (0.16)
5	1.61 (0.11)	-0.29(0.06)	0.89 (0.07)	1.51 (0.09)	2.38 (0.16)
6	1.09 (0.08)	-2.01(0.14)	-0.54(0.07)	0.34 (0.07)	2.07 (0.14)
7	1.62 (0.12)	-0.07(0.06)	0.94 (0.07)	1.63 (0.10)	2.57 (0.18)
8	1.04 (0.07)	-2.07(0.15)	-0.40(0.07)	0.70 (0.08)	1.98 (0.14)
9	1.21 (0.08)	-2.26(0.15)	-0.85(0.08)	0.24 (0.07)	1.39 (0.10)
10	1.34 (0.09)	-0.75(0.07)	0.67 (0.07)	1.63 (0.11)	3.25 (0.29)
11	1.52 (0.11)	-0.12(0.06)	0.83 (0.07)	1.35 (0.09)	2.40 (0.16)
12	2.17 (0.15)	-0.57(0.06)	0.30 (0.06)	0.94 (0.07)	1.70 (0.09)
13	0.50 (0.06)	0.01 (0.11)	2.02 (0.25)	3.75 (0.45)	6.30 (0.90)
14	0.90 (0.07)	-0.74(0.09)	0.45 (0.08)	1.22 (0.11)	2.72 (0.22)
15	1.43 (0.10)	-0.11(0.06)	0.91 (0.07)	1.71 (0.11)	2.78 (0.21)
16	1.32 (0.09)	-0.38(0.07)	0.71 (0.07)	1.42 (0.10)	2.60 (0.19)
17	1.45 (0.09)	-1.48(0.10)	-0.33(0.06)	0.60 (0.07)	1.75 (0.11)
18	0.86 (0.07)	-0.02(0.08)	1.22 (0.11)	1.85 (0.15)	3.55 (0.33)
19	0.78 (0.06)	-3.24(0.28)	-1.62(0.14)	-0.13(0.08)	1.45 (0.13)
20	1.23 (0.08)	-1.22(0.09)	0.02 (0.06)	0.81 (0.08)	2.15 (0.14)

*Notes.* *a*, $b_2$, $b_3$, $b_4$, and $b_5$ are the parameters of the model equation, where *a* denotes the discrimination parameter and $b_{u_i}$ denotes the threshold parameters. The standard errors are denoted as SE

Regardless of whether classical test theory or item response theory was applied, Items 3 and 13 consistently showed inadequate discrimination and difficulty parameters. These findings are consistent with those of previous studies based on classical test theory analyses (Nakashima et al., 2020; Sun et al., 2021). The poor performance of Items 3 and 13 may suggest a conceptual misalignment between the construct intended to be measured by the PI20 and the cognitive mechanisms underlying developmental prosopagnosia. Item 3 (“*I find it notably easier to recognize people who have distinctive facial features*”) is intended to capture the difficulty in recognizing faces lacking distinctive cues for individuals with developmental prosopagnosia (Duchaine et al., 2006; Yardley et al., 2008). Nevertheless, face identities of individuals with distinctive facial features are generally recognized more readily than those without such features (Smillie et al., 2024; Valentine, 1991); thus, even individuals without developmental prosopagnosia may also endorse this item at higher levels. Thus, responses to Item 3 may reflect general perceptual principles of face recognition rather than individual differences in face processing difficulties associated with developmental prosopagnosia. Therefore, revising Item 3 to directly ask about difficulties in recognizing faces without distinctive features would allow us to more directly assess the core nature of developmental prosopagnosia (Examples of minor changes, *“I find it more difficult than most people to recognize people without distinctive facial features”*). Furthermore, Item 13 (“*I am very confident in my ability to recognize myself in photographs*”) asks about the ability to recognize one’s own face. Previous studies have shown that individuals with developmental prosopagnosia commonly exhibit an own-face bias and demonstrate self-recognition performance comparable to that of controls (Malaspina et al., 2016, 2018, 2019). Individuals with developmental prosopagnosia have delayed event-related potentials to others’ faces compared to controls, but no difference is found for their own faces (Parketny et al., 2015). Even when some individuals with developmental prosopagnosia may still fail to recognize their own face, this characteristic may not be a strong item to screen for developmental prosopagnosia. Therefore, measuring true developmental prosopagnosia more accurately may require modifying the wording of these items, excluding them, or adding new items. For example, one approach is to replace “myself” with “my friends” to serve as a reversed item for the difficulty of recognizing familiar faces in Item 13. In summary, these findings highlight the need to reconsider how the PI20 measures the face processing difficulties of developmental prosopagnosia and may inform future refinements of its measurement structure.

This study has two limitations. The first limitation is that the participants and language were restricted to Japanese. The PI20 has been translated not only into Japanese (Nakashima et al., 2020) but also into various languages and is widely used worldwide (Estudillo & Wong, 2021; Nigrou et al., 2024; Nørkær et al., 2023; Sun et al., 2021; Tagliente et al., 2023; Ventura et al., 2018). In general, cross-linguistic and cultural differences in psychometric scales cannot be ruled out (van de Vijver and Tanzer, 2004), and their guidelines are inconsistent (Epstein et al., 2015). Linguistic equivalence has been examined with caution in several items of the PI20 (e.g., Mejia et al., 2026). Further investigation is needed to determine whether the present findings are consistent across languages and cultures, especially within the framework of classical test theory. The second limitation is that this study did not include behavioral measures beyond the PI20. Although numerous assessment tools exist for developmental prosopagnosia, a unified consensus has yet to be established (Nørkær et al., 2024; Susilo and Duchaine, 2025). Previous studies have supported the external validity of the PI20 through correlations with behavioral measures such as the Cambridge Face Memory Test and the Glasgow Face Matching Test (Shah et al., 2015a, b), but such associations were not directly examined in the present dataset. Self-report measures of developmental prosopagnosia have been shown to capture this condition to some extent, but they do not provide a definitive assessment (Arizpe et al., 2019; Matsuyoshi and Watanabe, 2021; Palermo et al., 2017). Given that several items of the PI20 showed suboptimal discrimination and difficulty parameters in the present analyses, it remains unclear whether excluding or revising such items would improve predictive accuracy when evaluated against behavioral measures. Investigating this issue with new data would facilitate closer alignment between self-report measures and the behavioral measures underlying developmental prosopagnosia.

The item-level insights obtained in this study provide a foundation for developing a revised version of the PI20 that maintains psychometric robustness while enhancing efficiency in research and clinical settings. This study is noteworthy for using both classical test theory and item response theory to evaluate the reliability and validity of the scale, while also pointing out issues with Items 3 and 13. Revising items that are less suitable for measuring developmental prosopagnosia is expected to reduce measurement artifacts and provide clinicians with a more practical screening tool than the current version. Therefore, revising such items can also facilitate large-scale epidemiological surveys by reducing participant burden. Beyond these practical implications, the present psychometric evaluation offers methodological insights for improving the reliability and validity of the PI20 in future research. By providing open-source code and data, the present study also facilitates independent and cross-sample validation of the PI20 across different populations, languages, and cultural contexts. Together, these findings provide a foundation for refining self-report assessments of developmental prosopagnosia and underscore the importance of rigorous psychometric evaluation in the development of screening instruments.

## Data Availability

The raw data is available as a file “MS_Oct20_2025_PI20_Data.csv” on the Open Science Framework (https://osf.io/qdjg2/).

## Code Availability

The R script is available as a file “MS_Oct20_2025_PI20_Program.txt” on the Open Science Framework (https://osf.io/qdjg2/).
